# Refractory Hypoxemia Secondary to Patent Foramen Ovale Presenting With Platypnea-Orthodeoxia Syndrome

**DOI:** 10.7759/cureus.111242

**Published:** 2026-06-21

**Authors:** Shanthan R Ramidi

**Affiliations:** 1 Hospital Medicine, Atrium Health, Concord, USA

**Keywords:** acute hypoxemic respiratory failure, patent forman ovale (pfo), refractory hypoxemia, stroke and hypoxemia, unexplained hypoxemia

## Abstract

Patent foramen ovale (PFO) is a common congenital cardiac anomaly present in 20%-25% of adults and is usually clinically silent. In rare cases, it can result in significant right-to-left shunting and refractory hypoxemia, particularly in platypnea-orthodeoxia syndrome (POS). We describe a 59-year-old woman with chronic hypoxemic respiratory failure who developed worsening oxygenation despite high-flow supplemental oxygen. Comprehensive evaluation excluded primary pulmonary, vascular, and parenchymal etiologies. Contrast echocardiography demonstrated a PFO with early right-to-left shunting. Percutaneous closure resulted in immediate and sustained improvement in oxygenation, with liberation from supplemental oxygen.

## Introduction

Most patent foramen ovales (PFOs) remain asymptomatic because left atrial pressure normally exceeds right atrial pressure, keeping the septal flap functionally closed and preventing significant blood flow between the atria [1]. A PFO becomes clinically significant when right atrial pressure transiently or persistently exceeds left atrial pressure, such as during a Valsalva maneuver, coughing, or conditions that elevate right-sided heart pressures, allowing right-to-left shunting [2]. Anatomical factors, including a large PFO or associated atrial septal aneurysm [3], can further increase shunt flow.

In rare circumstances, a PFO can result in significant right-to-left shunting, causing hypoxemia, particularly in the setting of altered intracardiac pressures or anatomical distortion of the interatrial septum [4]. Platypnea-orthodeoxia syndrome (POS) is a rare clinical entity characterized by dyspnea and arterial desaturation that worsen in the upright position and improve when supine. Although commonly associated with hepatopulmonary syndrome and pulmonary arteriovenous malformations, intracardiac shunting through a PFO remains an important and potentially treatable cause.

We report a case of severe refractory hypoxemia secondary to PFO-associated right-to-left shunting presenting with orthodeoxia in a patient with chronic cardiopulmonary disease.

## Case presentation

A 59-year-old woman with chronic hypoxemic respiratory failure on 5 L/min nasal cannula oxygen, paroxysmal atrial fibrillation, heart failure with improved ejection fraction status post implantable cardioverter-defibrillator (ICD) placement, type 2 diabetes mellitus, obesity, obstructive sleep apnea on continuous positive airway pressure (CPAP) therapy, hypertension, hypothyroidism, and hepatic steatosis presented with worsening dyspnea and hypoxemia. The patient had been admitted multiple times previously and was treated for congestive heart failure (CHF) exacerbations. She reported worsening dyspnea when sitting upright in a chair.

At home, her oxygen saturations had declined to 87%-88% despite adherence to her baseline oxygen therapy. On physical examination, her oxygen saturation remained in the low 90% range while receiving 8 L/min oxygen via nasal cannula in the supine position. Upon sitting upright, her oxygen requirements increased to 10-12 L/min, yet her oxygen saturation remained approximately 90%. Laboratory studies, including brain natriuretic peptide (BNP) and troponin levels, were unremarkable. Chest radiography and computed tomography pulmonary angiography demonstrated no acute cardiopulmonary abnormalities or evidence of pulmonary embolism.

The patient had experienced progressive exertional dyspnea and unexplained hypoxemia for several months. She was evaluated by Pulmonary Medicine as an outpatient, where pulmonary function testing revealed normal results. However, lower-extremity edema was noted, and she continued follow-up with Cardiology. Given her cardiac history, she was referred back to the Heart Failure team, and repeat right heart catheterization was performed. This demonstrated mildly elevated right atrial pressures (8-10 mmHg), normal pulmonary artery pressures, a reduced cardiac index of approximately 2 L/min/m², and an unobtainable pulmonary capillary wedge pressure (PCWP). Since then, her symptoms have persisted and progressively worsened, with increasing exertional dyspnea associated with oxygen desaturation and episodes of dizziness during activity.

Recent right heart catheterization demonstrated normal pulmonary artery pressures without evidence of pulmonary hypertension. Pulmonary function testing revealed preserved lung volumes and a normal diffusing capacity, with no evidence of restrictive lung disease.

Arterial blood gas analysis on 100% inspired oxygen demonstrated severe refractory hypoxemia with positional worsening, as shown in Table 1.

**Table 1:** Arterial blood gas analysis demonstrated positional hypoxemia, with a decline in PaO₂ from 64 mmHg in the supine position to 60 mmHg while upright, accompanied by mild respiratory alkalosis.

Position	pH	pCO_2_ (mmHg)	pO_2_ (mmHg)	HCO_3_- (mmol/L)
Supine	7.44	34	64	23
Upright	7.46	31	60	22

The failure of PaO₂ to improve despite maximal oxygen administration strongly suggested right-to-left shunt physiology. Positional worsening raised suspicion for POS.

**Figure 1:**
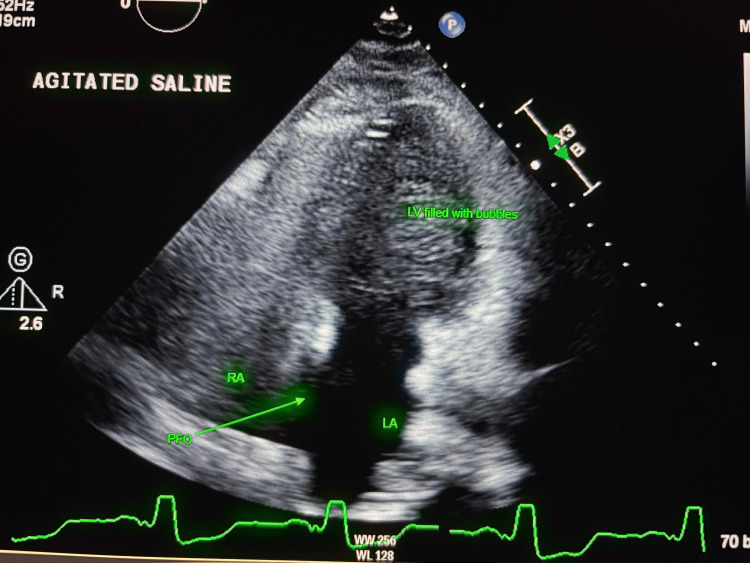
Apical four-chamber transthoracic echocardiographic view obtained during agitated saline (“bubble”) study demonstrating the LV filled completely with bubbles, indicating fairly large PFO. RA and LA are labeled for orientation. Appearance is consistent with evaluation for right-to-left interatrial shunting. LA: left atrium, LV: left ventricle, PFO: patent foramen ovale.

**Figure 2:**
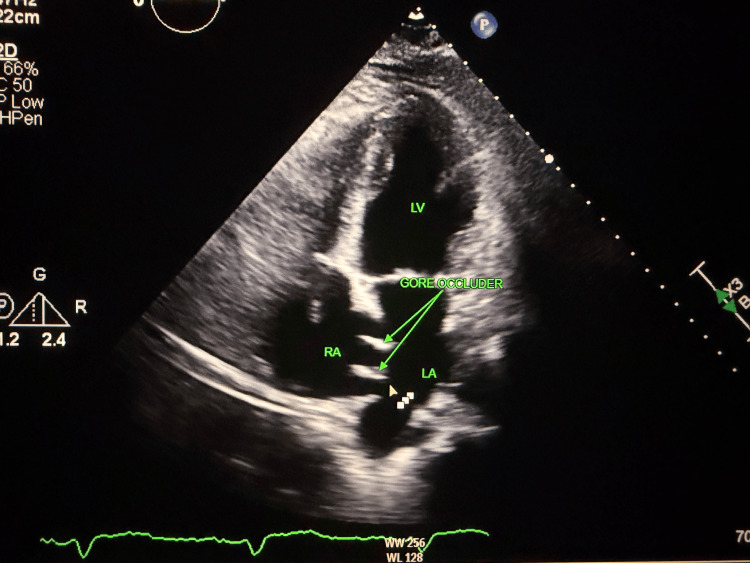
TTE After Deployment of a 30 mm Gore Occluder for PFO Closure TTE: transthoracic echocardiogram, PFO: patent foramen ovale.

Transthoracic echocardiography with agitated saline contrast demonstrated a PFO (Figure 1) with early right-to-left bubble transit consistent with intracardiac shunting. The patient underwent percutaneous PFO closure the following day, with rapid improvement in oxygenation (Figure 2). She was successfully weaned off supplemental oxygen and maintained an oxygen saturation of 98% on room air. At the time of discharge, she was saturating at 98% on room air. 

## Discussion

This case illustrates an uncommon but important cause of refractory hypoxemia due to intracardiac shunting through a PFO. PFO may cause clinically significant hypoxemia through right-to-left shunting, particularly in the presence of physiologic or structural factors that facilitate intracardiac flow despite normal right-sided pressures [5].

The patient demonstrated several features highly suggestive of shunt physiology, including profound hypoxemia refractory to 100% inspired oxygen, preserved pulmonary imaging, normal pulmonary artery pressures, absence of significant parenchymal lung disease, and positional desaturation. Failure of arterial oxygen tension to rise appropriately during administration of 100% inspired oxygen is a classic indicator of true right-to-left shunting rather than isolated ventilation-perfusion mismatch [3]. Under normal conditions, PaO₂ during 100% inspired oxygen administration typically exceeds 500 mmHg; therefore, a persistently low PaO₂ near 60 mmHg strongly supports substantial intracardiac shunting.

Our findings are consistent with prior reports describing POS associated with PFO. POS is characterized by worsening dyspnea and arterial desaturation in the upright position, with improvement when supine [6,7]. Several mechanisms have been proposed to explain positional shunting despite normal right-sided pressures, including age-related elongation or dilation of the aorta, distortion of the interatrial septum, preferential streaming of inferior vena cava blood toward the foramen ovale, and altered atrial geometry in the upright posture [6,7]. Similar to previously reported cases, our patient exhibited marked positional hypoxemia without evidence of pulmonary hypertension, emphasizing that elevated right atrial pressure is not required for clinically significant shunting [6,7].

The differential diagnosis initially included hepatopulmonary syndrome (HPS) because of the patient's hepatic steatosis and orthodeoxia. HPS is characterized by intrapulmonary vascular dilatation leading to positional hypoxemia and delayed appearance of bubbles in the left atrium during contrast echocardiography [8]. In contrast, the present case demonstrated early bubble transit across the interatrial septum, favoring intracardiac rather than intrapulmonary shunting. Previous studies have emphasized the utility of agitated saline contrast echocardiography in differentiating intracardiac from intrapulmonary causes of hypoxemia based on the timing of bubble appearance [8].

Recognition of PFO-associated hypoxemia is clinically important because definitive treatment with percutaneous closure can produce substantial symptomatic improvement and reduce oxygen requirements. Multiple observational studies and case series have reported marked improvement in arterial oxygenation and functional status following transcatheter PFO closure in patients with POS. Studies have demonstrated significant symptomatic benefit after closure in patients with positional hypoxemia related to PFO [9]. Early diagnosis is therefore essential, particularly in patients with unexplained refractory hypoxemia and preserved pulmonary imaging.

This case further highlights the importance of considering intracardiac shunting in the evaluation of severe hypoxemia that appears disproportionate to pulmonary findings. Failure to recognize this entity may delay definitive therapy and prolong unnecessary oxygen dependence.

## Conclusions

PFO should be considered in patients with unexplained refractory hypoxemia, particularly when associated with platypnea-orthodeoxia physiology and an unrevealing pulmonary evaluation. Severe hypoxemia despite 100% inspired oxygen strongly suggests right-to-left shunt physiology. Contrast echocardiography with agitated saline is essential for differentiating intracardiac from intrapulmonary shunting. This case also highlights the importance of maintaining a broad differential diagnosis in patients with chronic hypoxemia that appears disproportionate to the underlying pulmonary or cardiac disease. In patients initially presumed to have hypoxemia related to common conditions such as COPD or CHF, clinicians should remain alert to less common but potentially reversible etiologies, particularly when the clinical course, oxygen requirements, or diagnostic evaluation is atypical. Early recognition of PFO-associated hypoxemia is critical, as timely diagnosis and intervention may result in substantial symptomatic and functional improvement.
